# Prediction of the prognosis of liver iron burden within two years after hematopoietic stem cell transplantation based on multimodal MRI-based radiomics model

**DOI:** 10.3389/fmed.2025.1640625

**Published:** 2025-11-26

**Authors:** Fengming Xu, Suzhen Wei, Jixing Yi, Bumin Liang, Hanxiang Wei, Mengjun Huang, Haohua Wu, Qing Feng, Tao Wei, Tao Li

**Affiliations:** 1 Department of Radiology, Liuzhou Worker's Hospital, Liuzhou, China; 2 School of International Education, Guangxi Medical University, Nanning, China; 3 Department of Radiology, Liuzhou Municipal Liutie Central Hospital, Liuzhou, China; 4 Department of Radiology, Wuxuan County People's Hospital, Laibin, China; 5 Hepatobiliary Surgery Department, Liuzhou Worker's Hospital, Liuzhou, China

**Keywords:** radiomics, hematopoietic stem cell transplantation, thalassemia, MRI, model

## Abstract

To explore the predictive value of MRI-based radiomics model for the prognosis of liver iron burden within 2 years after hematopoietic stem cell transplantation (HSCT) in thalassemia (TM) patients who had undergone HSCT the preoperative liver 3.0T/1.5T MRI images and clinical data of 360 TM patients in two medical centers (A and B) were retrospectively analyzed. AUC, accuracy, sensitivity and specificity were used to evaluate the predictive efficacy of the model. The best performance prediction model of 3.0T/1.5T radiomics in medical center A was T1_F: the AUC, accuracy, sensitivity and specificity of the training set were 0.942/0.917, 0.91/0.8, 0.941/1 and 0.9/0.772, respectively. The AUC, accuracy, sensitivity and specificity of the test set were 0.845/0.896, 0.767/0.714, 1/1 and 0.696/0.667, respectively. The optimal performance prediction models of 3.0T/1.5T radiomics in medical center B were T1_W and T1_opp, respectively. The AUC, accuracy, sensitivity and specificity of the training set were 0.855/0.94, 0.79/0.933, 0.779/0.9 and 0.8/0.938, respectively. The AUC, accuracy, sensitivity and specificity of the test set were 0.81/0.743, 0.778/0.727, 0.73/0.727 and 0.73/0.714, respectively. It is expected that different MRI prediction models with different parameters can be constructed in different medical centers to evaluate the prognosis of liver iron burden in TM patients after HSCT.

## Introduction

Thalassemia (TM) is an inherited autosomal recessive blood disease caused by defects in globin chain production, which is caused by mutations in the globin gene or its regulatory region (1). The globin chain synthesis rate of hemoglobin in TM patients is reduced, resulting in the inability of the patient’s body to produce sufficient amounts of normal hemoglobin (2).

Regular blood transfusion, as a traditional treatment method, can correct the hemoglobin status and achieve temporary relief of anemia symptoms, but it cannot fundamentally cure TM (3, 4). At present, the only promising cure for this disease is Hematopoietic stem cell transplantation (HSCT) (2, 5). In the past two decades, with the development of strategies to control transplant-related complications and the reduction of toxicity associated with preparative regimens, the prognosis of some TM patients after transplantation has improved significantly (6). However, most TM patients received different frequencies of blood transfusion before and after receiving HSCT, and it takes a certain time for patients to reconstitute blood (from implantation to the beginning of recovery, and then to complete recovery) after HSCT (2, 7). Due to increased iron load secondary to ineffective erythropoiesis and enhanced intestinal absorption due to hepcidin deficiency, different degrees of iron load occur in the liver, heart, pancreas, and kidney of TM patients regardless of whether they have been treated with HSCT (2, 8). Although the most serious clinical complications caused by iron overload are caused by iron deposition in the heart, the liver, as the main iron storage organ, is the main manifestation of iron load in organs and the primary organ for clinical detection of iron load in organs (8). Therefore, it is necessary to monitor the liver iron loading status of TM patients (including those who have undergone HSCT within 2 years), and then adopt specific iron chelation therapy regimens for different patients for regular iron chelation treatment, so as to effectively prevent multiple organ diseases and premature death caused by iron overload (1, 2, 8). If the prognosis of liver iron burden in TM patients after receiving HSCT can be predicted as early as possible, it may be possible to make clinical suggestions earlier, so as to make corresponding preparatory diagnosis and treatment plans earlier, and intervene the prognosis of liver iron burden in time.

Magnetic Resonance Imaging (MRI) based imaging technology has become an important non-invasive examination for the diagnosis and evaluation of many diseases. The T2*, R*(1,000/T2*) technique of GRE imaging sequences has been established as a noninvasive standard for quantifying tissue iron content (9, 10). Due to its non-invasiveness, repeatability and high safety, this technique can achieve long-term and regular monitoring of iron content in organs of patients. However, this technique evaluates the degree of liver iron burden by measuring the immediate T2* and R2* values of the patient’s organs, which cannot realize the early prediction of liver iron burden in TM patients after HSCT. With the continuous development of medical imaging technology and radiomics, macroscopic image data can be deeply mined to reflect many high-level data that cannot be identified by the naked eye. Many studies have shown that radiomics technology can be applied to the differential diagnosis of many diseases, the prediction of tumor metastasis, the prediction of benign and malignant tumors, the evaluation of therapeutic effect, and the prognosis of diseases after treatment, and has shown good predictive performance (11–14).

The aim of this study is to construct a multi-parameter liver MRI radiomics model of TM patients before HSCT, and to explore the predictive value of the radiomics model for the prognosis of liver iron burden in TM patients within 2 years after HSCT, so as to provide an early prediction method for clinical practice and provide or change the corresponding diagnosis and treatment plan for patients as soon as possible.

## Materials and methods

### Research information

**Figure 1:**
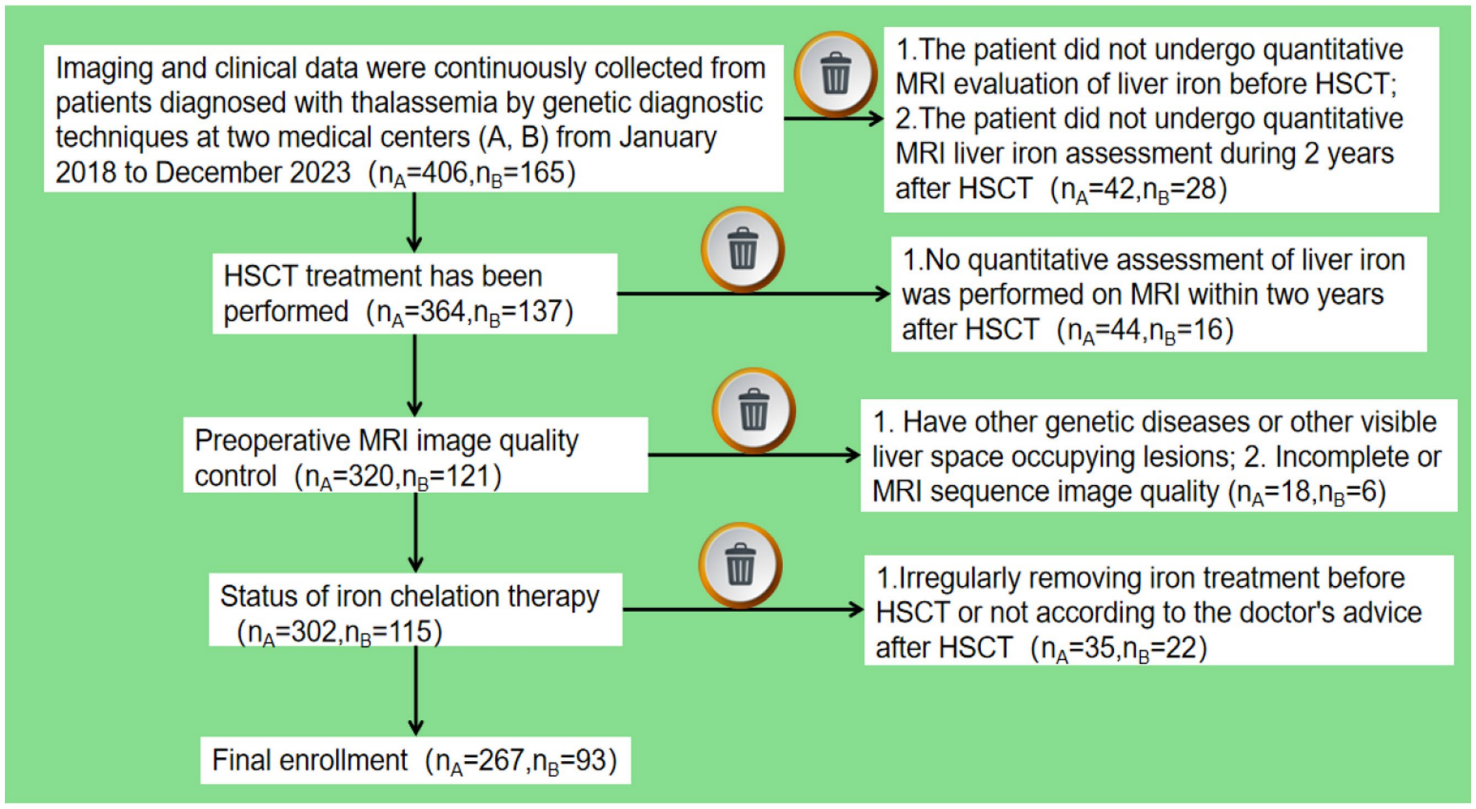
A simplified flow chart for the patient selection process.

The imaging and clinical data of 571 TM patients diagnosed by genetic diagnosis technology in two medical centers from January 2018 to December 2023 were continuously collected. Inclusion criteria: (1) undergoing HSCT, at least one time MRI T2* quantitative assessment of liver iron burden before HSCT and at least one time MRI T2* quantitative assessment of liver iron burden within 2 years after HSCT; (2) Complete preoperative liver MRI multi-parameter image sequence, including T2_fblade_fs(T2_fs), T1_vibe_dixon_tra_pre_opp/in/F/W(T1_opp/in/F/W); (3) regular or irregular blood transfusion; (4) Iron chelation therapy before after HSCT. Exclusion criteria: (1) combined with other genetic diseases or other macroscopic liver space-occupying lesions; (2) incomplete MRI sequence or poor image quality. Figure 1 shows the simplified workflow of inclusion and exclusion pathways in this study. Finally, A total of 267 and 93 TM patients from medical center A and B were included in this study.

### MRI scanning methods

A 3.0/1.5T MRI scanner (Center A: Philips Medical System, Amsterdam, The Netherlands; Center B: Philips Medical Systems, Best, The Netherlands) was used to perform liver scans (18-channel abdominal phased-array surface coil and 32-channel integrated spine matrix coil). The subjects were placed in the head-advanced, supine position. The GRE T2* sequence was acquired at the end of a single breath-hold expiration and single-layer sections were obtained in a single breath-hold at the maximum cross-sectional area of the liver. The remaining sequences (T2_fs, T1_opp/in/W/F) were scanned throughout the liver (see Table 1 for relevant parameters).

**Table 1:** MRI scan parameters.

Medical Center	Medical Center A		Medical Center B	
Field strength	3.0 T	1.5T	3.0 T	1.5T
T2*	TR = 5.8 ms, TE = 1.02, 1.72, 2.42, 3.12, 3.82, 4.52 ms; Flip angle = 3°, Matrix = 152 × 125, FOV = 375 mm × 312 mm, Layer thickness = 6 mm	TR = 6.5 ms, TE = 1.15, 1.95, 2.75, 355, 4.35, 5.15 ms; Flip angle = 5°, Matrix = 212 × 149, FOV = 375 mm × 297 mm, Layer thickness = 6 mm	TR = 200 ms, TE = 0.97, 2.38, 3.79, 5.20, 6.61, 8.02, 9.43, 10.84, 12.25, 13.66, 15.07, 16.48 ms; Flip angle = 20°, Matrix = 64 × 128, FOV = 200 mm × 400 mm, Layer thickness = 10 mm	TR = 200 ms, TE = 1.29, 2.35, 3.43, 4.6, 5.68, 6.85, 7.93, 9.1, 10.18, 11.35, 12.43, 13.6 ms; Flip angle 20°, Matrix = 256 × 256, FOV = 400 mm × 400 mm, Layer thickness = 10 mm
T2_fs	TR = 3,064 ms, TE = 89 ms; Flip angle = 90°, Matrix = 288 × 288, FOV = 400 mm × 400 mm, Layer thickness = 5 mm	TR = 1783 ms, TE = 80 ms; Flip angle = 90°, Matrix = 280 × 280, FOV = 380 mm × 380 mm, Layer thickness = 6 mm	TR = 3,110 ms, TE = 87 ms; Flip angle = 104°, Matrix = 384 × 384, FOV = 380 mm × 380 mm, Layer thickness = 5 mm	TR = 1829 ms, TE = 85 ms; Flip angle = 104°, Matrix = 380 × 380, FOV = 360 mm × 360 mm, Layer thickness = 6 mm
T1_opp/in/W/F	TR = 3.8 ms, TE = 1.32, 2.4 ms; Flip angle = 10°, Matrix = 288 × 225, FOV = 400 mm × 338 mm, Layer thickness = 5 mm	TR = 5.5 ms, TE = 1.73 ms, 3.7 ms; Flip angle = 10°, Matrix = 252 × 163, FOV = 400 mm × 325 mm, Layer thickness = 6 mm	TR = 6.3 ms, TE = 2.39, 4.77 ms; Flip angle = 9°, Matrix = 208 × 256, FOV = 308.8 mm × 380 mm, Layer thickness = 4.5 mm	TR = 7.5 ms, TE = 2.67, 4.80 ms; Flip angle = 9°, Matrix = 212 × 149, FOV = 338 mm × 400 mm, Layer thickness = 5.5 mm

### Clinical characteristic analysis

A total of 267 and 93 patients with TM from Medical Centers A and B, respectively, were included in the study. The blood routine, liver and kidney function of 78 and 81 patients were collected from Medical Center A and B, respectively. The collected liver T2 * values were graded according to the severity of clinical liver iron load: 1.5T grade [severe group (< 1.4 ms), moderate group (1.4–2.7 ms), mild group (2.7–6.3 ms), normal group (> 6.3 ms)]; 3T grade [severe group (< 1.59 ms), moderate group (1.59–2.17 ms), mild group (2.17–3.78 ms), normal group (> 3.78 ms)] (13, 14). According to whether T2 * increased by 50% or whether the severity of liver iron load improved within 24 months after HSCT (for example, severe to moderate, mild or normal; Moderate to mild or normal; Mild to normal), it was divided into two groups: the prognosis of liver iron load after HSCT was better (positive group, 1) and the prognosis of liver iron load was worse (negative group, 0).

All clinical data were analyzed by SPSS 26.0 statistical software package. All statistical tests were two-sided, and *p* < 0.05 was considered statistically significant. The measurement data were expressed as mean, standard deviation (x ®± s), median (M) and quartile (P_25%_-P_75%_). Count data were expressed by the number of cases and percentile (%). Binary logistic regression was used to analyze the blood routine, liver and kidney function characteristics of TM patients in the two medical centers, and the clinical characteristics with statistical significance were retained (*p* < 0.05), and a clinical model was constructed.

## Radiomics analysis

### Construction of radiomics prediction model

The MRI images of TM patients in the two medical centers included: (1) two kinds of field strengths at 1.5T and 3T; (2) Five modes: T2_fs, T1_opp, T1_in, T1_F, T1_W. A total of 20 radiomics models were constructed (the model in center A was recorded as A3T-T2_fs, A3T-T1_opp/in/F/W, A1.5T-T2_fs, A1.5T-T1_opp/ IN /F/W; The model constructed in center B was denoted as B3T-T2_fs, B3T-T1_opp/in/F/W. B1.5T-T2_fs, B1.5T-T1_opp/in/F/W). In DICOM format from PACS workstation, images of TM patients were uploaded to Darwin Research platform (Version 3.3.9, http://10.100.118.3:8089), which is launched by Yizhun Medical AI technology limited company, which is an artificial intelligence research platform for medical imaging (Refer to https://arxiv.org/pdf/2009.00908v1). The image-based feature extraction, machine learning model development, and statistical data analysis was carried out on Darwin Research platform. Within this platform, the scikit-learn (0.22.2.post1) and PyTorch (2.9.0.post0) libraries are used to implement the integration method. The MRI data were tuned using the ComBat method homogenization program based on the empirical Bayesian model correction method built into the Darwin research platform.

Radiomics feature extraction followed the guidelines of the Imaging Biomarker Standardization Initiative (IBSI) to ensure repeatability and standardization (15, 16). This includes standardized image preprocessing, extracting features from the delineated volume of interest, and applying the IBSI nomenclature to all reported features. Specifically: 1. Image preprocessing: All images are resampled to isotropic 1 × 1 × 1 mm^3^ voxels using B-spline interpolation. 2. Feature extraction: A total of 1,125 features were extracted from each delineated volume of interest. Its mathematical definitions and calculations strictly follow the IBSI reference manual. 3. Nomenclature: All extracted features are reported using the standardized IBSI nomenclature to facilitate cross-study comparisons. Imageomics analysis included the following steps: (1) Region of interest (ROI) two-dimensional segmentation: all parametric images were selected for manual segmentation of the maximum liver cross section (Appendix E1). (2) Feature extraction: shape features, first-order features and texture features. Shape features describe the basic geometric characteristics of the scratched area, including size, shape and surface roughness. First-order features use common and fundamental metrics to describe the distribution of voxel intensities within the delineated region. Texture features include gray level co-occurrence Matrix (GLCM), gray level free length Matrix (GLRLM), gray level size region Matrix (GLSZM), adjacent gray level color difference Matrix (NGTDM) and gray level dependence Matrix (GLDM). These features are able to capture the spatial interdependence of voxels in an image and show the spatial heterogeneity features of the image, such as gray level variation, spacer size, and roughness. In addition, six filters including exponential, square, square root, log, log-sigma-3-0-MM-3D and wavelet were used to further process the first-order features and texture features. The wavelet filter extracts features from eight wavelet decomposed images. A total of 1,125 features were extracted. (3) Radiomics feature selection and model construction: the data from the two medical centers were randomly divided into A training set (medical center A, 187 cases; Medical center B, 65 cases) and validation set (medical center A, 80 cases; Medical center B, 28 cases). All radiomic features were normalized using minimum–maximum values to eliminate the magnitudes of different features by scaling values to [0, 1]. The sample variance *F* value (f_classif) was used to screen out 20 important features for classification. In this study, two methods were used to select significant radiomics features for the training cohort from feature subsets of different parametric MRI sequences, alone or in combination. These methods include least absolute shrinkage and selection operator (LASSO) and Logistic Regression (LR). LASSO selected the relevant features according to the best parameter (alpha). LR was used to select the optimal features. In the construction of the LR model in this study: K = 5 in k-fold cross validation. The evaluation index was roc_auc. The elasticnet is selected as the penalty parameters, with the penalty coefficient C = 1 and the l1_ratio = 0.5. And the maximum number of retained features. On this basis, machine learning models are built based on the selected features and logistic regression. The Rad score constructed for each patient by the logistic regression classifier will help predict the prognosis of liver iron burden in TM patients after HSCT.

### Performance of Imagomics prediction models

Calibration curves were used to evaluate whether the model prediction probabilities were close to the true probabilities. The receiver operating characteristic (ROC) curve, area under the curve (AUC), Accuracy, Sensitivity and Specificity were used to comprehensively evaluate the prediction performance of the model.

### Intra-observer and inter-observer consistency

Intra-observer and inter-observer consistency in feature extraction was assessed by intraclass correlation coefficient (ICC). A total of 25T2_fs images were randomly selected for ROI segmentation and feature extraction. ROI segmentation was performed independently by two experienced radiologists (both with more than 5 years of experience in abdominal MRI diagnosis and proficient in the Darwin Research platform). The intra-observer ICC was calculated by comparing the features (10 features selected using random numbers) extracted by observer A twice. The inter-observer ICC was calculated by comparing the features extracted by observer B with those extracted by observer A. When ICC > 0.75 and *p* < 0.05, the consistency was considered good. When the inter-observer agreement was good, all image segmentation and feature extraction tasks were averaged and randomly assigned to observers A and B.

## Results

### Clinical features

A total of 267 TM patients from Medical center A were enrolled in this study (median age, 10.04 years; inter-quartile range [8.17–12.08 years]; 99 females (37.08%); 168 males (62.92%). A total of 93TM patients from Medical center B were enrolled in this study (median age, 9.00 years; inter-quartile range [6.00–11.00 years]; 44 females (47.31%); 49 males (52.69%) were included in this study. After multivariate logistic regression screening, the data of blood routine, liver and kidney function of TM patients in the two medical centers had no significant statistical significance in predicting the efficacy of liver iron burden after HSCT, so this study did not construct clinical regression, and only presented as baseline data (Table 2).

**Table 2:** Baseline data before HSCT in TM patients from medical center A/B.

Variables	Medical centre A					Medical centre B				
	Mean	Median	Std. Deviation	Minimum–Maximum	P25%-P75%	Mean	Median	Std. Deviation	Minimum–Maximum	P25%-P75%
WBC	5.74	5.15	2.09	2.30–10.91	4.23–6.78	12.21	6.85	35.64	0.08–319.30	4.87–9.83
RBC	3.78	3.83	0.51	2.50–5.23	3.42–4.07	4.19	4.33	1.21	0.14–6.88	3.40–5.00
HGB	103.03	104.00	15.07	71.00–147.00	92.00–113.00	106.93	110.00	28.72	3.00–157.00	90.35–125.50
PLT	282.95	261.50	122.37	28.00–733.00	197.00–373.00	250.39	181.00	216.41	0.80–1291.00	111.30–307.60
NEU%	0.42	0.43	0.11	0.11–0.72	0.33–0.50	0.49	0.47	0.18	0.07–0.96	0.37–0.59
LYM%	0.48	0.48	0.11	0.25–0.85	0.40–0.57	0.37	0.39	0.16	0.01–0.61	0.28–0.49
MONO%	0.07	0.07	0.02	0.01–0.17	0.05–0.08	0.09	0.08	0.06	0.00–0.35	0.07–0.11
EO%	0.02	0.02	0.02	0.00–0.15	0.01–0.03	0.03	0.03	0.03	0.00–0.16	0.01–0.04
NEU	2.45	2.26	1.15	0.25–6.59	1.63–3.19	4.03	3.00	4.54	0.06–25.81	1.97–4.44
LYM	2.72	2.52	1.15	1.28–7.73	1.97–3.05	3.85	2.78	6.16	0.00–36.76	1.47–3.57
MONO	0.41	0.36	0.21	0.05–1.14	0.27–0.48	0.88	0.50	1.90	0.01–16.74	0.34–0.81
EOS	0.13	0.11	0.17	0.00–1.40	0.04–0.17	0.25	0.16	0.36	0.00–2.31	0.06–0.26
BISO	0.03	0.02	0.02	0.00–0.09	0.01–0.03	1.10	0.02	9.60	0.00–86.46	0.01–0.04
MCV	80.93	81.65	3.96	69.40–89.40	78.38–83.80	77.59	81.25	11.38	39.58–101.20	68.05–85.80
MCH	27.28	27.60	1.67	23.60–31.00	25.88–28.40	31.26	26.72	48.20	17.60–457.80	21.79–29.57
MCHC	337.03	336.00	10.21	304.00–365.00	330.00–344.50	326.42	329.80	41.39	0.06–374.00	317.00–343.15
HCT	0.31	0.31	0.04	0.21–0.43	0.28–0.33	0.40	0.34	0.68	0.01–6.39	0.29–0.39
RDW	0.37	0.16	1.77	0.12–15.70	0.13–0.19	0.17	0.15	0.06	0.11–0.56	0.13–0.19
PDW	46.11	44.00	7.86	35.60–66.70	40.05–50.35	0.14	0.13	0.05	0.08–0.41	0.09–0.17
PCT	10.53	10.10	2.14	7.70–18.10	9.30–10.90	0.22	0.15	0.19	0.01–1.20	0.10–0.28
MPV	0.00	0.00	0.00	0.00–0.01	0.00–0.00	8.72	8.80	2.15	0.16–23.14	7.86–9.48
TBiL	19.92	16.40	9.60	6.40–47.60	12.90–25.30	14.07	9.50	27.53	2.50–251.70	6.20–14.65
DBiL	7.97	7.30	3.06	3.00–17.60	5.63–9.98	6.21	3.10	21.13	1.20–191.40	2.45–4.80
IBil	11.99	9.40	7.07	3.30–35.50	6.60–15.20	7.86	6.10	7.31	0.10–60.30	3.70–10.40
DB/TB	0.76	0.72	0.26	0.34–1.69	0.63–0.83	0.37	0.34	0.14	0.19–0.96	0.30–0.43
TP	69.60	70.65	9.56	1.30–82.00	66.40–73.95	68.84	68.20	7.47	52.90–95.00	64.20–72.75
ALB	43.98	43.30	3.33	34.20–51.40	41.85–46.05	39.72	40.10	3.28	30.20–47.00	38.10–42.20
GLO	25.27	25.30	5.39	0.61–35.90	22.40–28.58	29.12	28.40	7.35	11.50–63.00	25.05–32.25
A/G	1.81	1.80	0.37	1.10–2.90	1.60–2.00	1.45	1.40	0.43	0.50–3.70	1.20–1.55
GGT	15.59	13.00	12.07	5.00–74.00	9.00–16.00	69.20	22.00	247.46	8.00–1733.00	14.00–38.50
TBA	9.97	6.45	14.22	0.80–89.90	4.10–10.75	18.03	5.70	39.15	0.92–259.80	2.30–14.25
AST	28.99	23.00	18.46	4.11–106.00	19.00–31.00	40.62	31.00	35.76	9.00–300.00	24.00–47.00
ALT	29.82	21.00	30.46	2.00–184.00	11.00–33.00	39.38	24.00	51.72	6.00–387.00	18.00–41.50
AST/ALT	1.42	1.20	0.98	0.40–7.50	0.90–1.60	1.34	1.30	0.61	0.30–3.40	0.90–1.60
ALP	235.69	215.00	93.77	2.80–542.00	182.00–285.50	233.57	216.00	133.26	69.00–927.00	146.00–298.00
CHE	6909.69	6,766	1641.22	3,269–10,987	5,679–7,635	7762.02	7,561	2422.77	3,392–16,085	6,115–9,284
UREA	5.67	5.40	1.80	2.70–11.50	4.30–6.63	5.47	5.03	2.19	2.26–14.55	3.99–6.52
CREA	33.97	33.00	10.40	17.00–64.00	25.00–40.00	31.20	30.00	9.97	12.00–71.00	25.00–36.00
UA	259.50	269.00	86.31	97.00–446.00	181.25–328.25	247.77	257.00	89.51	42.00–414.00	195.50–310.00
ccr	107.21	94.00	47.36	57.00–333.00	82.00–117.00	86.48	82.10	23.18	38.00–179.00	69.75–99.00
CysC	0.86	0.88	0.25	0.24–1.48	0.70–1.01	0.94	0.93	0.24	0.44–1.90	0.78–1.09
RBP	24.79	25.00	6.64	13.00–39.00	20.00–29.00	32.76	32.85	11.58	12.70–81.50	24.60–38.00

WBC, white blood cell (10^9/L); RBC, red blood cell (10^12/L); HGB, hemoglobin (g/L); PLT, Platelets (10^9/L); NEU%, neutrophile granulocyte%; LYM%, lymphocyte%; MONO%, mononuclear cells%; EO%, eosinophile granulocyte%; BISO%, basophile granulocyte%; NEU, neutrophile granulocyte; LYM, lymphocyte (10^9/L); MONO, mononuclear cells (10^9/L); EOS, eosinophile granulocyte (10^9/L); BISO, basophile granulocyte (10^9/L); MCV, mean corpuscular volume (fL); MCH, Mean Corpuscular Hemoglobin (pg); MCHC, Mean Corpuscular Hemoglobin Concentration (g/L); HCT, hematocrit; RDW, red cell distribution width; PDW, platelet distribution width; PCT, Platelet cubic measure distributing width; MPV, Mean Platelet Volume; TBiL, total bilirubin (μmol/L); DBiL, Direct Bilirubin (μmol/L); IBil, Indirect Bilirubin (μmol/L); DB/TB, Direct Bilirubin/Indirect Bilirubin; TP, Total protein (g/L); ALB, Albumin; GLO, Globulin; A/G, Albumin/Globulin; GGT, gamma-glutamyl transpeptidase (U/L); TBA, Total Bile Acid (μmol/L); AST, Aspartate Aminotransferase (U/L); ALT, Alanine Aminotransferase (U/L); AST/ALT, Aspartate Aminotransferase/Alanine Aminotransferase; ALP, A Lkaline Phosphatase (U/L); CHE, cholinesterase (U/L); CREA, Creatinine (μmol/L); UA, Uric Acid (μmol/L); ccr, Creatinine Clearance Rate (ml/min); CysC, Cystatin from chicken egg white (Cmg/L); RBP, Retinol Binding Protein (mg/L).

### Radiomics features

**Figure 2:**
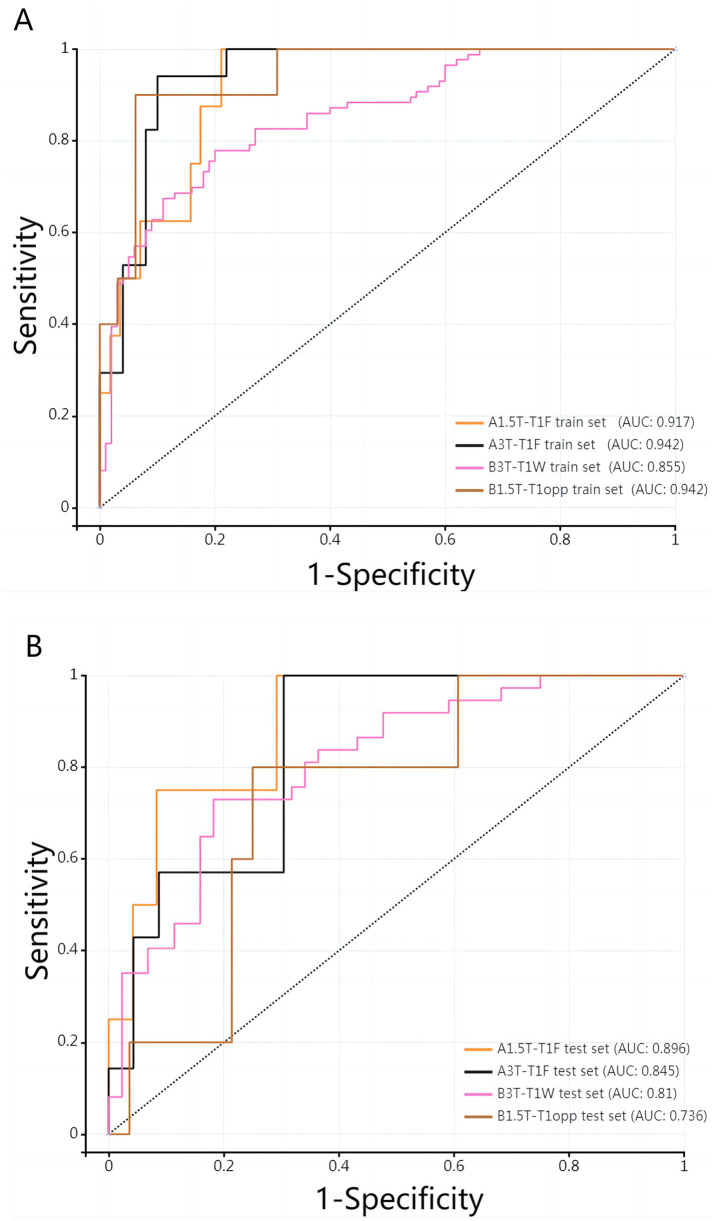
**(A,B)** ROC curves of the best 1.5T/3T radiomics prediction model in the two medical centers.

**Figure 3:**
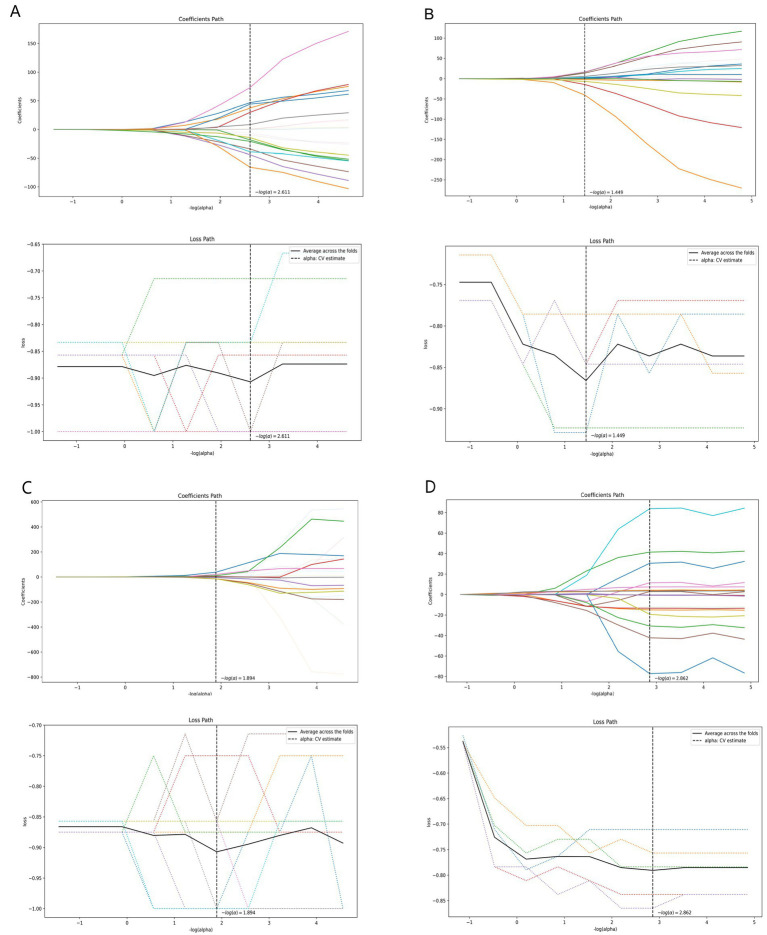
Radiomics feature dimensionality reduction LASSO coefficient distribution map (**A–D** correspond to A1.5T-T1_F, A3T-T1_F, B1.5T1_opp, B3T1_W models respectively). Each curve represents the change trajectory of the independent variable coefficient corresponding to different penalty coefficients, and the dotted line is the minimum penalty coefficient.

**Figure 4:**
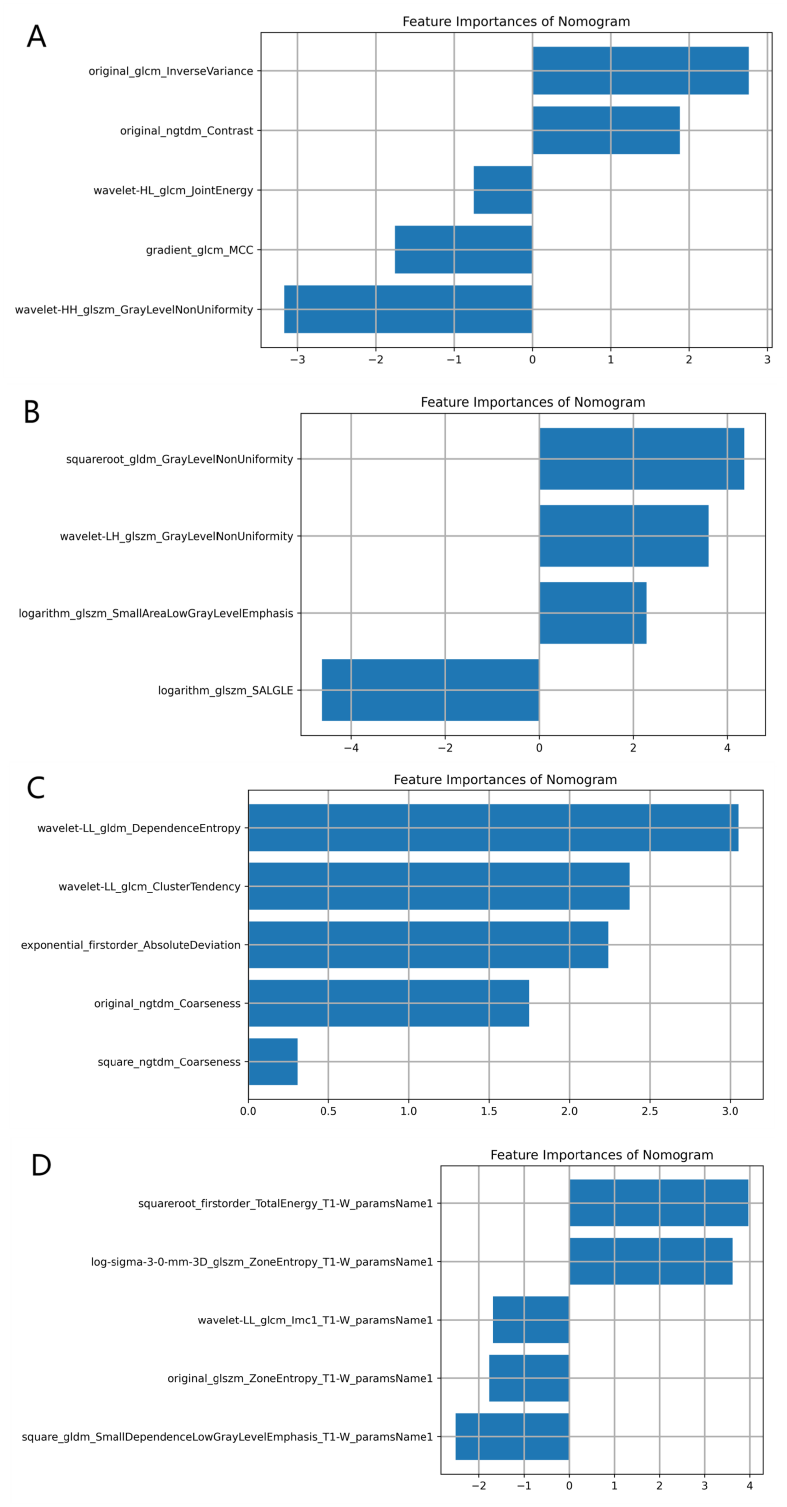
Visualization of feature importance for different models. **(A–D)** Represent the feature importance visualization of A1.5T-T1_F, A3T-T1_F, B1.5T1_opp, and B3T1_W, respectively. Due to the different MRI scanning parameters in different centers, the optimal features selected for the construction of the prediction model are also different. However, in the same model, different features have different importance for prediction performance.

**Figure 5:**
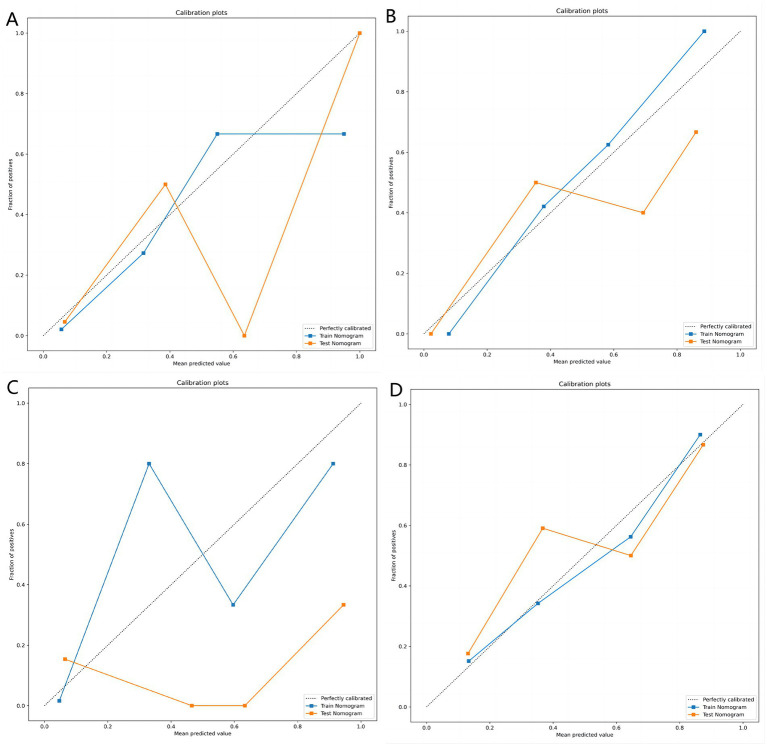
**(A–D)** Represent the calibration curves of the matching degree between the predicted results and the actual results of the A1.5T-T1_F, A3T-T1_F, B1.5T1_opp, and B3T1_W prediction models, respectively. Obviously, the A3T-T1_F and B3T1_W models showed better prediction effect.

**Figure 6:**
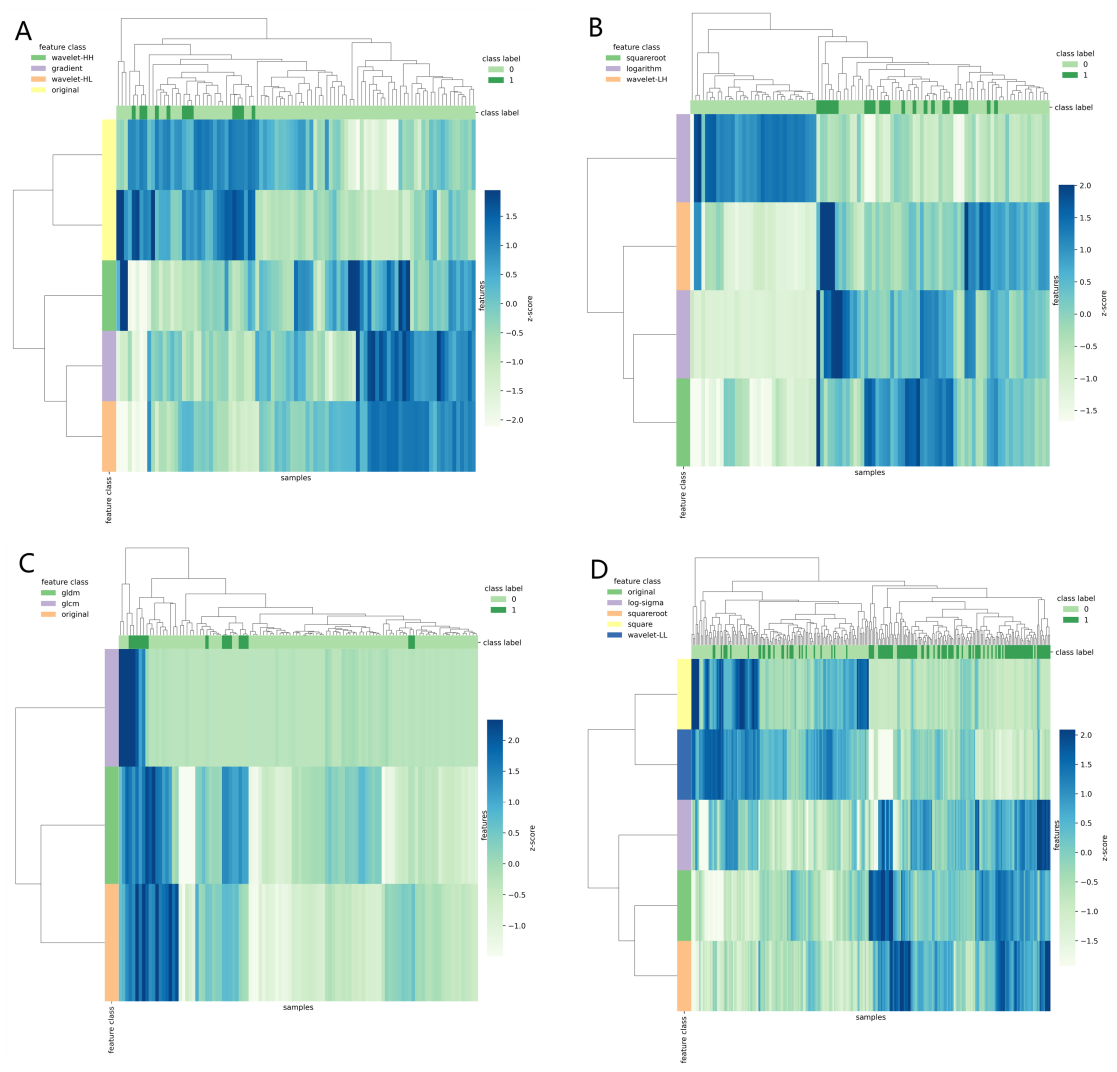
Clustering heat map (**A–D** correspond to A1.5T-T1_F, A3T-T1_F, B1.5T1_opp, B3T1_W models respectively) used different colors to reflect the value of each one-dimensional feature (row) of the sample (column). The top color bar expresses the true class of the samples, the left color bar expresses the class of the features, and the dendrogram shows the results of the hierarchical clustering. By comparing the clustering results, real labels and heat maps, it is helpful to control the data quality and intuitively show the difference of feature distribution in different categories.

**Figure 7:**
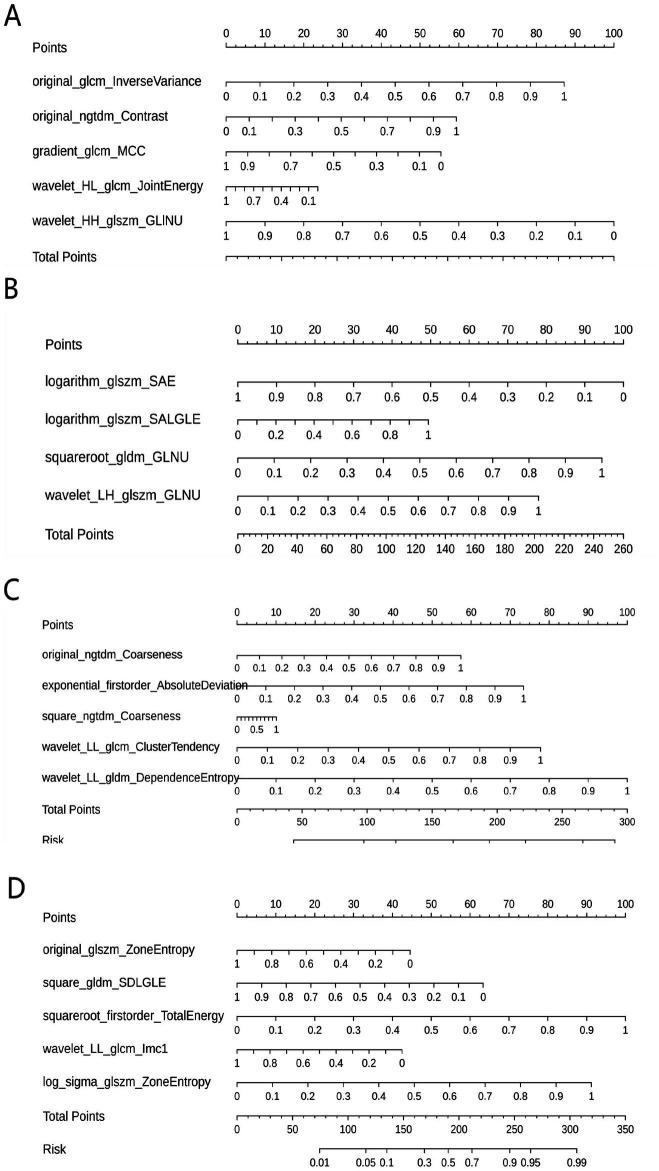
Nomograms of different prediction models. (**A–D** correspond to A1.5T-T1_F, A3T-T1_F, B1.5T1_opp, B3T1_W models respectively). The corresponding Rad scores of different prediction models are: Rad Score (A1.5T-T1_F) = −3.169 × wavelet-HH_glszm_GrayLevelNonUniformity + 2.763 × original_glcm_InverseVariance + 1.881 × original_ngtdm_Contrast—1.756 × gradient_glcm_MCC-0.749 × wavelet-HL_glcm_JointEnergy-2.440; Rad Score (A3T-T1_F) = −4.618 × logarithm_glszm_SmallAreaEmphasis + 4.360 × squareroot_gldm_GrayLevelNonUniformity + 3.602 × wavelet-LH_glszm_GrayLevelNonUniformity + 2.281 × logarithm_glszm_SmallAreaLowGrayLevelEmphasis—3.902; Rad Score (B1.5T1_opp) = + 5.266 × original_gldm_DependenceEntropy_paramsName1 + 2.689 × exponential_glcm_ClusterTendency_paramsName1 + 2.095 × original_ngtdm_Coarseness—6.420; RadScore (B3T1_W) = + 3.968 × squareroot_firstorder_TotalEnergy_paramsName1 + 3.621 × log-sigma-3-0-mm-3D_glszm_ZoneEntropy_paramsName1–2.515 × square_gldm_SmallDependenceLowGrayLevelEmphasis_paramsName1–1.768 × original_glszm_ZoneEntropy_paramsName1–1.686 × wavelet-LL_glcm_Imc1_paramsName1–1.583. GLINU = GrayLevelNonUniformity, SAE = SmallAreaEmphasis, SALGLE = SmallAreaLowGrayLevelEmphasis, GLNU = GrayLevelNonUniformity, SDLGLE = SmallDependenceLowGrayLevelEmphasis.

Table 3 shows the diagnostic performance of radiomics models with different MRI modalities in the two medical centers. The best 1.5T and 3T radiomics models in center A were T1_F. In A1.5T-T1F training group and test group, AUC, Sensitivity, Specificity and Accuracy were 0.917 (0.841, 0.992)/0.896 (0.747, 1), 1 (0.676, 1)/1 (0.51, 1), 0.772 (0.648, 0.862)/0.667 (0.467, 0.82), 0.8/0.714, respectively. In A3T-T1F training group and test group, AUC, Sensitivity, Specificity and Accuracy were 0.942 (0.89, 0.995)/0.845 (0.697, 0.992), 0.941 (0.73, 0.99)/1 (0.646, 1), 0.9 (0.786, 0.957)/0.696 (0.491, 0.844), 0.91/0.767, respectively. The best 1.5T imaging radiomics model of center B was T1 _ opp. The AUC, Sensitivity, Specificity and Accuracy of B1.5T-T1_opp training group and test group were 0.942 (0.876, 1)/0.736 (0.517, 0.954), 0.9 (0.596, 0.982)/0.8 (0.376, 0.964), 0.938 (0.852, 0.976)/0.714 (0.529, 0.847), 0.933/0.727, respectively. The best 3T imaging omics model is T1 _ W, and the AUC, Sensitivity, Specificity and Accuracy of the B3T-T1 _ W training group and test group are 0.794 (0.729, 0.859)/0.693 (0.576, 0.811), 0.773 (0.675, 0.848)/0.658 (0.499, 0.788), 0.724 (0.629, 0.803)/0.698 (0.549, 0.814), 0.747/0.679, respectively, (the ROC curve is shown in Figure 2, this article only shows the relevant pictures of these four models). The 20 different radiomics models had different diagnostic efficiencies, indicating that the prediction models constructed by different centers, different field strengths and different modes had different predictive efficiencies in predicting the prognosis of liver iron burden within 2 years after HSCT in TM patients. Figure 3 shows the process of selecting the optimal radiomics features of different models using LASSO and alpha. Four radiomics features were included in A1.5T-T1_F and A3T-T1_F, respectively, and five radiomics features were included in B1.5T1_opp and B3T1_W, respectively. The visualization of the importance of the features used is shown in Figure 4. The prediction effects of different prediction models are shown in Figure 5. A3T-T1_F and B3T1_W had better prediction effects in training group and test group. However, the prediction effect of A1.5T-T1_F in the training group was better, and the prediction effect of the validation group was slightly worse. (Figure 6) The cluster heatmaps of four different models showed a significant difference between good and poor prognosis of liver iron burden in TM patients within 2 years after HSCT. By comparing the clustering results, real labels and heat maps, it is helpful to control the data quality and intuitively show the difference of feature distribution in different categories. Figure 7 shows the different model nomograms and their corresponding Rad scores.

**Table 3:** Evaluation table of diagnostic efficacy of different modality MRI radiomics models in medical center A/B.

Model	Medical centre A				Medical centre B			
	AUC (95%CI)	Sensitivity (95%CI)	Specificity (95%CI)	Accuracy	AUC (95%CI)	Sensitivity (95%CI)	Specificity (95%CI)	Accuracy
1.5T-T1_F_train	0.917 (0.841, 0.992)	1 (0.676, 1)	0.772 (0.648, 0.862)	0.8	0.78 (0.697, 0.864)	0.919 (0.787, 0.972)	0.579 (0.467, 0.684)	0.69
1.5T-T1_F_test	0.896 (0.747, 1)	1 (0.51, 1)	0.667 (0.467, 0.82)	0.714	0.723 (0.582, 0.865)	0.875 (0.64, 0.965)	0.515 (0.352, 0.675)	0.633
1.5T-T1_W_train	0.858 (0.714, 1)	0.625 (0.306, 0.863)	0.983 (0.91, 0.997)	0.94	0.809 (0.729, 0.888)	0.868 (0.727, 0.942)	0.584 (0.473, 0.688)	0.678
1.5T-T1_W_test	0.77 (0.554, 0.986)	1 (0.51, 1)	0.52 (0.335, 0.7)	0.586	0.601 (0.424, 0.779)	0.25 (0.102, 0.495)	1 (0.898, 1)	0.76
1.5T-T1_opp_train	0.94 (0.872, 1)	0.9 (0.596, 0.982)	0.938 (0.852, 0.976)	0.933	0.942 (0.876, 1)	0.9 (0.596, 0.982)	0.938 (0.852, 0.976)	0.933
1.5T-T1_opp_test	0.743 (0.555, 0.931)	0.8 (0.376, 0.964)	0.714 (0.529, 0.847)	0.727	0.736 (0.517, 0.954)	0.8 (0.376, 0.964)	0.714 (0.529, 0.847)	0.727
1.5T-T1_in_train	0.848 (0.762, 0.934)	0.739 (0.535, 0.875)	0.781 (0.673, 0.86)	0.771	0.777 (0.675, 0.88)	0.583 (0.422, 0.729)	0.918 (0.832, 0.962)	0.807
1.5T-T1_in_test	0.8 (0.663, 0.937)	1 (0.722, 1)	0.531 (0.364, 0.691)	0.643	0.61 (0.444, 0.777)	0.533 (0.301, 0.752)	0.688 (0.514, 0.82)	0.638
1.5T-T2_fs_train	0.838 (0.746, 0.93)	0.609(0.408, 0.778)	0.93 (0.846, 0.97)	0.851	0.78 (0.697, 0.864)	0.919 (0.787, 0.972)	0.579 (0.467, 0.684)	0.69
1.5T-T2_fs_test	0.639 (0.453, 0.824)	0.9 (0.596, 0.982)	0.419 (0.264, 0.592)	0.537	0.723 (0.582, 0.865)	0.875 (0.64, 0.965)	0.515 (0.352, 0.675)	0.633
3T-T1_F_train	0.942 (0.89, 0.995)	0.941 (0.73, 0.99)	0.9 (0.786, 0.957)	0.91	0.794 (0.729, 0.859)	0.773 (0.675, 0.848)	0.724 (0.629, 0.803)	0.747
3T-T1_F_test	0.845 (0.697, 0.992)	1 (0.646, 1)	0.696 (0.491, 0.844)	0.767	0.693 (0.576, 0.811)	0.658 (0.499, 0.788)	0.698 (0.549, 0.814)	0.679
3T-T1_W_train	0.87 (0.782, 0.958)	0.867 (0.621, 0.963)	0.75 (0.628, 0.842)	0.773	0.794 (0.729, 0.859)	0.773 (0.675, 0.848)	0.724 (0.629, 0.803)	0.747
3T-T1_W_test	0.79 (0.589, 0.992)	1 (0.61, 1)	0.481 (0.307, 0.66)	0.576	0.693 (0.576, 0.811)	0.658 (0.499, 0.788)	0.698 (0.549, 0.814)	0.679
3T-T1_opp_train	0.816 (0.725, 0.907)	0.87 (0.679, 0.955)	0.644 (0.529, 0.744)	0.698	0.793 (0.728, 0.857)	0.848 (0.761, 0.907)	0.687 (0.59, 0.77)	0.764
3T-T1_opp_test	0.753 (0.572, 0.934)	0.9 (0.596, 0.982)	0.625 (0.453, 0.771)	0.69	0.792 (0.696, 0.888)	0.725 (0.572, 0.839)	0.762 (0.615, 0.865)	0.744
3T-T1_in_train	0.848 (0.762, 0.934)	0.739 (0.535, 0.875)	0.781 (0.673, 0.86)	0.771	0.782 (0.716, 0.848)	0.793 (0.7, 0.864)	0.663 (0.562, 0.751)	0.728
3T-T1_in_test	0.8 (0.663, 0.937)	1 (0.722, 1)	0.531 (0.364, 0.691)	0.643	0.805 (0.707, 0.903)	0.825 (0.681, 0.913)	0.725 (0.572, 0.839)	0.775
3T-T2_fs_train	0.838 (0.746, 0.93)	0.609 (0.408, 0.778)	0.93 (0.846, 0.97)	0.851	0.824 (0.766, 0.883)	0.804 (0.712, 0.873)	0.713 (0.614, 0.794)	0.758
3T-T2_fs_test	0.639 (0.453, 0.824)	0.9 (0.596, 0.982)	0.419 (0.264, 0.592)	0.537	0.729 (0.615, 0.842)	0.65 (0.495, 0.779)	0.829 (0.687, 0.915)	0.741

### Inter-observer and intra-observer reproducibility of radiomics feature extraction

Based on the two measurements of observer A, the intra-observer ICC ranged from 0.848 to 0.988 (*p* < 0.001). Based on the measurements of observers A/B, the inter-observer agreement ranged from 0.842 to 0.988 (p < 0.001). The results showed that the intra-observer and inter-observer feature extraction had good consistency.

## Discussion

In this study, 20 liver MRI radiomics models were developed to predict the prognosis of liver iron burden in TM patients after HSCT. The results showed that the prediction performance of radiomics models in different centers was different due to the different MRI scanning parameters. The prediction performance of the same modality radiomics model in the same center may vary due to different magnetic fields. However, only for the current research sample, the two medical centers were able to construct radiomics models under different magnetic fields and different modes, which showed excellent prediction performance. These results indicate that the machine learning model can be regarded as a non-invasive tool to help predict the prognosis of liver iron burden in TM patients within 2 years after HSCT.

For clinical factors, multivariate logistic regression analysis showed that there were no independent factors with high value in predicting the prognosis of liver iron load within 2 years after HSCT in TM patients. This may be due to the fact that most TM patients received different frequency of blood transfusion before and after HSCT, and after HSCT, blood reconstruction takes a certain time (2, 7, 17), resulting in irregular blood routine and other factors. Therefore, no relevant clinical prediction model was constructed in this study, and the clinical data were only presented as baseline data.

For radiomics features, a total of 1,125 selected features including first-order, second-order and higher-order features were extracted using Darwin’s scientific research platform for in-depth exploration. Different radiomics features can describe or reflect different information. For example, order features can quantitatively describe the distribution of voxels in an image. The characteristics of gray level co-occurrence Matrix can reflect the homogeneity and heterogeneity of lesions. The feature of gray-scale run-length Matrix can reflect the directionality and roughness of image texture (18). In this study, the sample variance *F* value (f_classif) and the iteration after LASSO regression were used to screen out the best features to improve the accuracy of the classifier and its performance on high-dimensional datasets. In addition, logistic regression employing the constructed prediction model is a related supervised learning method for classification and regression, which can easily account for the influence of each input feature on the output result. In order to confirm the value of radiomics models in predicting the prognosis of liver iron burden in TM patients within 2 years after HSCT, and to avoid the chance of a single model, a total of 20 radiomics models from different centers and different field strengths (1.5T/3T) MRI were constructed in this study. For the imaging data of the two medical centers, different radiomics models had different prediction performance. The best prediction model at 1.5T/3T for center A was T1_F model; The T1_opp model and T1_W model were the best prediction models for center B at 1.5T and 3T, respectively. Although the model is not a unified and universal model, it is sufficient to show that different medical centers can construct different radiomics models to predict the prognosis of liver iron burden in TM patients within 2 years after HSCT. It is worth noting that although high-field MRI can better display tissues (19), after comparing the prediction models of the same sequence in the same center, we found that the diagnostic efficiency of the 3T prediction model is not always higher than that of the 1.5T prediction model of the same sequence.

Although this study constructed 20 radiomics models based on data from two centers to predict the prognosis of liver iron burden in TM patients within 2 years after HSCT, and some of the models showed high diagnostic efficiency, this study had the following limitations: (1) The analysis was based on data from only two medical centers without cross-validation or external validation. However, it is well known that radiomics feature values are significantly affected by technical Settings such as imaging equipment and scanning parameters. However, these effects are more heterogeneous in MR Studies, that is, without the same MR Scanning parameter scheme, for the same patient, the same body area of the same patient, and the image obtained on the same scanner, it also lacks tissue specificity and absolute intensity numerical significance (20). Nevertheless, although a unified and universal radiomics model cannot be constructed by independently modeling in different centers and different modalities in this study, it is enough to prove that different centers can indeed construct their own unique radiomics model to predict the prognosis of liver iron burden in TM patients within 2 years after HSCT. (2) In the process of constructing the model, a fixed random number was selected. In fact, a variety of random numbers were selected in the process of constructing the model in this study, and it was indeed found that different random numbers had different degrees of influence on the results of constructing the model. However, since the predictive performance of 20 different radiomics models has been listed in this study, the author believes that the effect of random number is not enough to deny the value of the radiomics model in predicting the prognosis of liver iron burden within 2 years after HSCT in TM patients. (3) Due to the heavy workload, this study did not build a predictive model through other machine learning methods (such as support vector machines, etc.). Therefore, future work should be to expand the sample size, balance the dataset and validate the model based on more central data. As far as possible, under the condition of unified multi-center MR Scanning parameters, the model construction of multiple machine learning methods was compared, and then a unified general prediction model was constructed for clinical reference. (4) The main reason for adopting the two-dimensional (2D) manual ROI method in this study is to be consistent with the established clinical practice and widely used methods in the field of liver iron quantification, especially techniques such as the R2* relaxation method (14). Many pioneering and clinical validation studies (9, 10) have placed manual 2D roi in uniform areas of the liver, avoiding large blood vessels and bile ducts, as a standard approach. By adhering to this practice, the results of this study ensure direct comparability with existing literature and current clinical benchmarks. Furthermore, from a practical perspective, the 2D manual ROI method has significant advantages in terms of feasibility and reproducibility among observers, especially in multi-center Settings. Three-dimensional volume segmentation of the entire liver is time-consuming, requires specialized software, and the repeatability among different institutions and operators may be variable. The 2D method of this study enables effective and standardized data analysis in all patient cohorts, minimizing the possibility of inter-observer variability that might be introduced by more complex 3D segmentation. It is undeniable that a complete 3D volume analysis can provide a more detailed assessment and is an exciting direction for future technological development. However, based on the purpose of this study, we believe that the strictly applied roi 2D method provides a reliable, clinically relevant, and reproducible estimation of liver iron storage.

In summary, despite the limitations of this study, this study finally demonstrated the predictive value of radiomics models in predicting the prognosis of liver iron burden in TM patients within 2 years after HSCT by constructing multi-modality radiomics prediction models. Different medical centers are expected to build unique radiomics prediction models based on their specific MR Scanning protocols, so as to evaluate the prognosis of liver iron burden in TM patients after HSCT in advance, and formulate, prepare and change the corresponding diagnosis and treatment plan as soon as possible.

## Data Availability

The original contributions presented in the study are included in the article/supplementary material, further inquiries can be directed to the corresponding authors.

## Appendix E1

Region of interest (ROI) two-dimensional segmentation: all parametric images were selected for manual segmentation of the maximum liver cross section.
